# Shen Shuai II recipe improves renal hypoxia to attenuate renal injury in 5/6 renal ablation/infarction rats and effect evaluation using blood oxygenation level-dependent functional magnetic resonance imaging

**DOI:** 10.1080/0886022X.2024.2338565

**Published:** 2024-04-15

**Authors:** Yizeng Xu, Fang Lu, Meng Wang, Lingchen Wang, Chaoyang Ye, Shuohui Yang, Chen Wang

**Affiliations:** a Department of Nephrology, Shuguang Hospital Affiliated to Shanghai University of Traditional Chinese Medicine, Shanghai, China; b Key Laboratory of Liver and Kidney Diseases, Ministry of Education, Shanghai University of Traditional Chinese Medicine, Shanghai, China; c TCM Institute of Kidney Disease, Shanghai University of Traditional Chinese Medicine, Shanghai, China; d Shanghai Key Laboratory of Traditional Chinese Clinical Medicine, Shanghai University of Traditional Chinese Medicine, Shanghai, China; e Department of Radiology, Shuguang Hospital Affiliated to Shanghai University of Traditional Chinese Medicine, Shanghai, China; f Department of Radiology, Shanghai Municipal Hospital of Traditional Chinese Medicine, Shanghai University of Traditional Chinese Medicine, Shanghai, China

**Keywords:** Shen Shuai II recipe, chronic kidney disease, renal hypoxia, blood oxygenation level-dependent functional magnetic resonance imaging

## Abstract

**Background:** Renal hypoxia plays a key role in the progression of chronic kidney disease (CKD). Shen Shuai II Recipe (SSR) has shown good results in the treatment of CKD as a common herbal formula. This study aimed to explore the effect of SSR on renal hypoxia and injury in CKD rats. **Methods:** Twenty-five Wistar rats underwent 5/6 renal ablation/infarction (A/I) surgery were randomly divided into three groups: 5/6 (A/I), 5/6 (A/I) + losartan (LOS), and 5/6 (A/I) + SSR groups. Another eight normal rats were used as the Sham group. After 8-week corresponding interventions, blood oxygenation level-dependent functional magnetic resonance imaging (BOLD-fMRI) was performed to evaluate renal oxygenation in all rats, and biochemical indicators were used to measure kidney and liver function, hemoglobin, and proteinuria. The expression of fibrosis and hypoxia-related proteins was analyzed using immunoblotting examination. **Results:** Renal oxygenation, evaluated by BOLD-fMRI as cortical and medullary T2* values (COT2* and MET2*), was decreased in 5/6 (A/I) rats, but increased after SSR treatment. SSR also downregulated the expression of hypoxia-inducible factor-1α (HIF-1α) in 5/6 (A/I) kidneys. With the improvement of renal hypoxia, renal function and fibrosis were improved in 5/6 (A/I) rats, accompanied by reduced proteinuria. Furthermore, the COT2* and MET2* were significantly positively correlated with the levels of creatinine clearance rate (Ccr) and hemoglobin, but negatively associated with the levels of serum creatinine (SCr), blood urea nitrogen (BUN), serum cystatin C (CysC), serum uric acid (UA), 24-h urinary protein (24-h Upr), and urinary albumin:creatinine ratio (UACR). **Conclusion:** The degree of renal oxygenation reduction is correlated with the severity of renal injury in CKD. SSR can improve renal hypoxia to attenuate renal injury in 5/6 (A/I) rats of CKD.

## 1. Introduction

Chronic kidney disease (CKD), as a progressive deterioration of renal function, remains a major public health problem with significant morbidity around the world [1]. Regardless of the initial etiology, activation of fibroblasts and deposition of extracellular matrix induce renal fibrosis and promote the progression of CKD [2]. Chronic hypoxia is the main pathological factor of renal fibrosis and considered as a common pathway to end-stage renal failure [3]. Owing to the inefficient oxygen delivery of renal arterial-to-venous diffusion shunt, renal oxygenation is relatively lower in physiological state, which we define as the unique borderline hypoxia [4]. Furthermore, the pathological milieus as CKD progression, such as inflammation, anemia and proteinuria, also aggravates renal hypoxia [5]. Thus, decreased renal oxygenation could be an independent predictor for assessing renal injury in CKD, and effective evaluation methods and treatments are required to avoid hypoxic injury.

In the theory of traditional Chinese medicine (TCM), the basic pathological feature of CKD is the spleen-kidney deficiency, and almost accompanied with dampness, heat, qi stagnation, and blood stasis [6]. Shen Shuai II Recipe (SSR), a common Chinese herbal formula, has been widely used for the treatment of CKD as an effective remedy for more than 20 years in our hospital. The properties of SSR are including tonifying the spleen and kidney, clearing heat and dampness, and activating blood circulation to resolve stasis. Our previous experimental studies have demonstrated that SSR has potential anti-inflammatory and antifibrotic effects under renal hypoxia condition [7–8]. Additionally, we found that SSR could improve renal perfusion and reduce renal oxygen consumption in 5/6 renal ablation/infarction (A/I) rats [9]. Therefore, the renoprotective effect of SSR may be associated with the improvement of renal oxygen supply.

Blood oxygenation level-dependent functional magnetic resonance imaging (BOLD-fMRI) provides a noninvasive evaluation of renal oxygenation by deoxyhemoglobin paramagnetic characteristics, and the result can be reflected by intrinsic tissue MR parameters (the effective transverse relaxation time T2* and the effective transverse relaxation rate R2*, R2* = 1/T2*) [10–11]. A lower T2* value (or higher R2* value) refers to greater concentration of deoxyhemoglobin, which indicates more severe tissue hypoxia. Owing to the advantages of noninvasion and convenience, BOLD-fMRI is regarded as a promising technique for assessing renal hypoxia, and the diagnostic value of it has been demonstrated in several studies of kidney diseases. Liwen Zhang et al. [12] confirmed that BOLD-fMRI can dynamically evaluate intrarenal oxygenation in mice with lupus nephritis. The feasibility of BOLD-fMRI in evaluating renal hypoxia in diabetic kidney disease has also been demonstrated in clinical and experimental studies [13–14]. Prasad et al. [15] supported the feasibility of BOLD-fMRI to quantitatively assess renal oxygen availability in the clinical application. In our clinical trials, we found that cortical and medullary T2* values (COT2* and MET2*) were declined with the progression of CKD [16–17]. Hence, the purpose under experimental research was to explore whether SSR could improve renal hypoxia to alleviate renal injury in a rat model of CKD, and further demonstrate the correlation of renal oxygenation with renal function.

## 2. Materials and methods

### 2.1. Animals and drugs

The animal study was approved by the Animal Experiment Ethics Committee of Shanghai University of Traditional Chinese Medicine (SHUTCM) (PZSHUTCM220725008), and all Wistar rats were obtained from SLAC Laboratory Animal Co., Ltd. (License No. SYXK 2022-0012). The experimental animal center of SHUTCM provides standard laboratory environment and diet for the rats. Nine raw herbs of SSR, shown in Table 1, were purchased from Shanghai Kangqiao Chinese Medicine Tablet Co., Ltd (Shanghai, China). As a positive control, losartan tablets (100 mg/tablet) were acquired from Merck Sharp & Dohme Pharmaceutical Co., Ltd. (Hangzhou, China).

**Table 1.** Composition and information of SSR.

Herb	Chinese name	Medicinal part	Quantities (g)
Codonopsis Radix	Dang shen	Rhizome	15
Epimedii Folium	Yin yang huo	Leaf	15
Salviae Miltiorrhizae Radix et Rhizoma	Dan shen	Root and rhizome	15
Angelicae Sinensis Radix	Dang gui	Root	15
Prepared Rhei Radix et Rhizoma	Da huang	Root and rhizome	15
Perillae Folium	Zi su ye	Leaf	15
Chuanxiong Rhizoma	Chuan xiong	Rhizome	15
Persicae Semen	Tao ren	Seed	15
Coptidis Rhizoma	Huang lian	Rhizome	6

### 2.2 Preparation of SSR and identification of major components

The SSR was prepared by the Department of Pharmacy, Shuguang Hospital Affiliated to SHUTCM for experiment *in vivo* as previously described, and the production methods were as follows [18]: the above nine herbs were proportionately combined with distilled water and decocted twice (one hour each time) with constant stirring. The aqueous extract was centrifuged for supernatant and purified with ethanol overnight at 4 °C. After removing ethanol by rotary evaporation, the supernatant was collected and dried using a freeze dryer.

The major chemical components of SSR, identified by Ultra-High Performance Liquid Chromatography-High Resolution Mass Spectrometry, was reported in our previous study [7], including rosmarinic acid (21.280 mg/each gram of SSR), amygdalin (6.588 mg/g), salvianolic acid B (1.852 mg/g), caffeic acid (1.390 mg/g), icariin (1.055 mg/g), berberine hydrochloride (0.503 mg/g), codonopsis lactone (0.130 mg/g), ferulic acid (0.127 mg/g), emodin (0.111 mg/g), and tanshinone IIA (0.005 mg/g).

### 2.3. Animal study protocol

**Figure 1.**
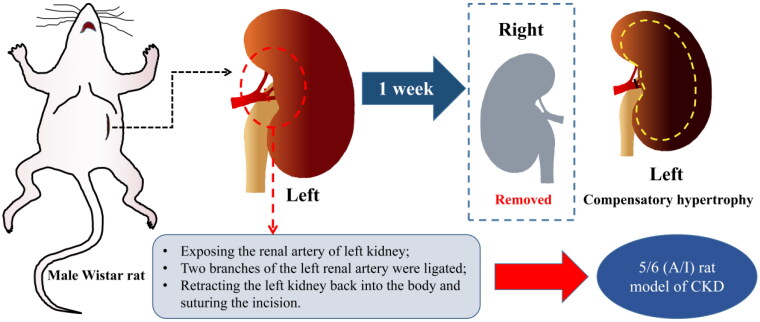
Construction of the 5/6 (A/I) rat model of CKD. 5/6 (A/I), 5/6 renal ablation/infarction; CKD, chronic kidney disease.

CKD was induced in rat by 5/6 renal ablation/infarction (A/I) surgery as we have previously reported [8,19], the surgical procedure was shown in Figure 1. Four weeks after surgery, 25 successfully modeled rats were under blood biochemistry tests including liver and kidney function and blood routine. Subsequently, 25 CKD rats and eight control rats were randomly divided into four groups: 5/6 (A/I) group (*n* = 8), 5/6 (A/I) + SSR group (*n* = 9), 5/6 (A/I) + Losartan (LOS) group (*n* = 8), and the sham operation (Sham) group (*n* = 8). The 8-week continuous daily administration of SSR (10 mL/kg/d, containing 4 g/ml of the original drug) and Losartan (10 mL/kg/d, the concentration of 5 mg/mL) were determined according to the previous study [20]. Additionally, the 5/6 (A/I) and Sham groups were gavaged with the same amount of distilled water.

After eight weeks of intervention, total 24-h urine of each rats was collected by metabolic cage to detect 24-h urinary protein (24-h Upr), urinary albumin:creatinine ratio (UACR). Renal oxygenation was evaluated by BOLD-fMRI in all rats. Blood routine, liver and kidney function were tested at the same time. Creatinine clearance rate (Ccr) was also calculated to assess glomerular filtration rate: Ccr (mL/min) = urine creatinine (μmol/L) × 24-h urine volume (mL)/[serum creatinine (μmol/L) × 1440 (min)] [9]. Finally, all the rats were euthanized with sodium pentobarbital (150 mg/kg, i.p.), and their kidneys were obtained for histological and molecular studies.

### 2.4. MRI protocol

**Figure 2.**
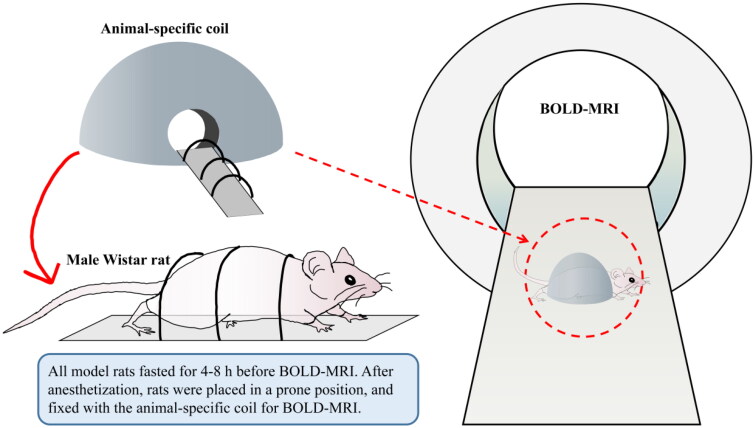
Flowchart of BOLD-fMRI examination in rats. BOLD-fMRI, blood oxygenation level-dependent functional magnetic resonance imaging.

**Figure 3.**
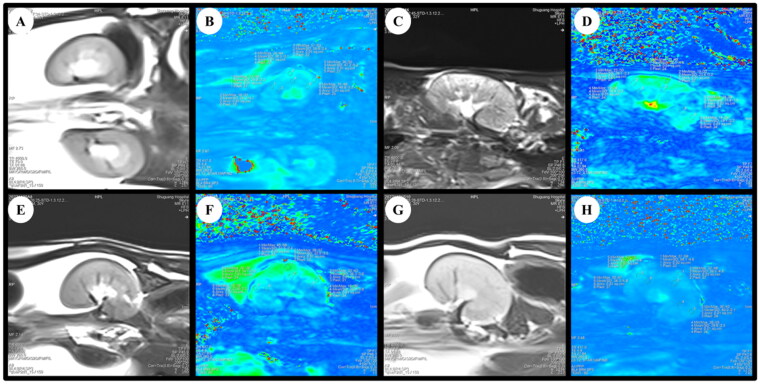
BOLD-fMRI T2*WI and T2* map in the coronal plane of rats. (A, B) The rat from Sham group. (C, D) The rat from 5/6 (A/I) group. (E, F) The rat from 5/6 (A/I) + LOS group. (G, H) The rat from 5/6 (A/I) + SSR group. ROI-based technique, with placement of circled ROIs in the renal cortex and medulla. BOLD-fMRI, blood oxygenation level-dependent functional magnetic resonance imaging; ROI, region of interest.

The rats were fasted for 4–6 h before MRI scanning. After anesthetization with 2% pentobarbital sodium (40 mg/kg, i.p.), rats were placed in a prone position and fixed with the animal-specific coil (Figure 2). The BOLD-fMRI examination was performed on a 3.0 T magnetic resonance scanner (MAGNETOM Skyra; Siemens Healthcare, Erlangen, Germany) as our previous clinical study described [16]. All the rats underwent routine T1-weighted imaging (T1WI), T2-weighted imaging (T2WI), and T2*WI (BOLD) imaging for all kidneys using a coronal multi-echo (5 echoes) gradient-echo sequence with echo times of 4.36, 11.90, 19.44, 29.68, and 34.52 ms; a repetition time of 417 ms; a voxel size of 0.2 mm × 0.2 mm × 2 mm; a flip angle of 60°; a bandwidth of 260 Hz/Px; a field of view of 120 mm; a 205 × 256 matrix, and the time of scanning was 47 s. T2WI* maps for each kidney were automatically generated in line immediately after the acquisitions, cortical and medullary T2* values (COT2* and MET2*) were evaluated separately by two experienced radiologists using six regions of interest (ROIs) with 0.01 cm^2^ placed at the upper, middle and lower areas of the central slice, avoiding vessels, renal sinuses and susceptibility artifacts carefully (Figure 3(A–H)). COT2* and MET2* were acquired from the left kidneys in all rats.

### 2.5. Histopathological examination

The removed kidneys were immediately fixed in 4% paraformaldehyde, embedded in paraffin, and sectioned into 3 μm slices. The sections were subjected to hematoxylin-eosin (HE) and Masson’s trichrome staining according to the standard protocol, and then observed under the microscope (Nikon Eclipse 80i, Japan) at 200 × magnification. For HE-stained sections, tubular injury was semiquantitatively scored as follows [21]: Score 0, no tubular injury; Score 1, < 25% of tubules injured; Score 2, 25–50% of tubules injured; Score 3, 51–75% of tubules injured; Score 4, > 75% of tubules injured. The severity of renal fibrosis was estimated by the % fibrotic area in four randomly selected fields per Masson’s trichrome-section of kidney using ImageJ v.1.53 (National Institutes of Health, USA).

### 2.6. Western blot analysis

The remnant kidney tissues were ground and lysed, and proteins were extracted using RIPA lysis buffer containing protease and phosphatase inhibitor. Equal amounts of protein samples, quantified by the BCA method, were separated on sodium dodecyl sulfate polyacrylamide gels and electrotransferred to polyvinylidene difuoride (PVDF) membranes. Incubating with blocking buffer for 1 h, the PVDF membranes were incubated with anti-HIF-1α (1:1000, A11945, Abclonal), anti-FN (1:2000, ab45688, Abcam), anti-α-SMA (1:2000, ab5694, Abcam), anti-Col-I (1:2000, ab260043, Abcam), and anti-GAPDH (1:2000, 60004-1-Ig, Proteintech) overnight at 4 °C. Next, the membrane was incubated with secondary antibodies (1:1000, Proteintech) for 1 h. The signals were detected and visualized with the Luminescent Imaging Workstation (Tanon, China), and the images were quantified using ImageJ v.1.53 (National Institutes of Health, USA).

### 2.7. Statistical analysis

Analyses were performed with SPSS 26.0 and GraphPad Prism 9.3. Normal distributions for continuous variables were evaluated by the Shapiro–Wilk test. All data were presented as mean ± SE and analyzed by one-way analysis of variance (ANOVA), Kruskal–Wallis H test and Student’s *t*-test as appropriate. The reproducibility of BOLD-fMRI was tested by the intraclass correlation coefficient (ICC). The relationships between BOLD-fMRI parameters (COT2* and MET2*) and biochemical indicators were analyzed by the Pearson correlation coefficient. A two-tailed *p* value < 0.05 was considered to be statistically significant.

## 3. Results

### 3.1. Reproducibility of BOLD-fMRI in rats

**Figure 4.**
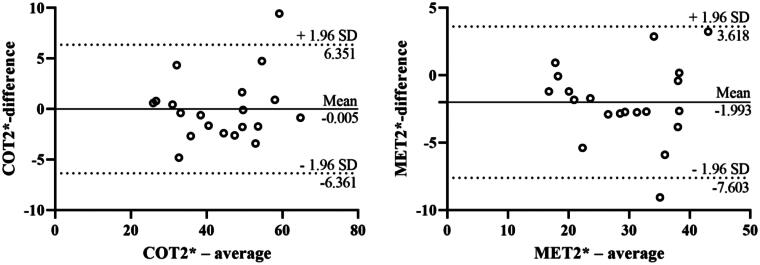
Bland-Altman plots of BOLD-fMRI analyses between observers. BOLD-fMRI, blood oxygenation level-dependent functional magnetic resonance imaging.

BOLD-fMRI evaluations were successfully completed in the rats. In order to evaluate the inter-observer variability of the classical ROI-based method, the BOLD-fMRI data of 20 rats were randomly enrolled and separately analyzed by two radiologists with 20 and 16 years’ experience of MR image interpretation. The reproducibility of both COT2* (ICC = 0.961) and MET2* (ICC = 0.941) were high. On Bland-Altman analyses, the mean differences and 95% limits of agreement (1.96 SD) for COT2* and MET2* were −0.005 (-6.361 to 6.351), −1.993 (-7.603 to 3.618) respectively (Figure 4).

### 3.2. SSR improves renal hypoxia in 5/6 (a/I) rats

**Figure 5.**
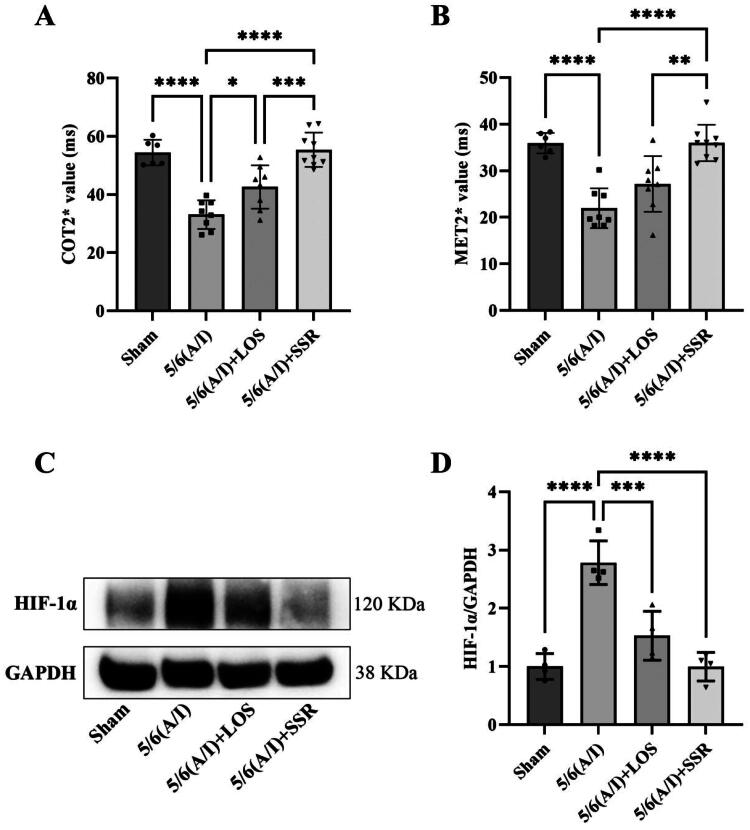
Effects of SSR on renal oxygenation in 5/6 (A/I) rats. (A) The level of COT2* was evaluated by BOLD-fMRI after treatment. (B) The level of MET2* was evaluated by BOLD-fMRI after treatment. (C) The expression of HIF-1α protein was determined by western blot. (D) Quantitative analysis of HIF-1α protein level (*n* = 4). Data were analyzed by One-way ANOVA. Values are mean ± SE. *p < 0.05; **p < 0.01; ***p < 0.001; ^****^p < 0.0001. COT2*, cortical T2*; MET2*, medullary T2*; LOS, Losartan; SSR, Shen Shuai II Recipe.

As shown in Table 2 and Figure 5(A–B), COT2* were significantly higher than MET2* in all rats in four groups (*t* = 15.911, *p* < 0.0001). The COT2* and MET2* in 5/6 (A/I) group were significantly lower than that in Sham group (‘33.11 ± 4.95 ms’ vs. ‘54.43 ± 4.37 ms’, *p* < 0.0001; ‘21.95 ± 4.28 ms’ vs. ‘35.94 ± 2.20 ms’, *p* < 0.0001). The 8-week treatment with SSR significantly improved renal hypoxia, reflected by increased COT2* and MET2*, in 5/6 (A/I) rats (‘55.39 ± 5.89 ms’ vs. ‘33.11 ± 4.95 ms’, *p* < 0.0001; ‘35.99 ± 3.92 ms’ vs. ‘21.95 ± 4.28 ms’, *p* < 0.0001), and the efficacy of SSR was superior to losartan (‘55.39 ± 5.89 ms’ vs. ‘42.60 ± 7.43 ms’, *p* < 0.001; ‘35.99 ± 3.92 ms’ vs. ‘27.15 ± 5.98 ms’, *p* < 0.01). Furthermore, we evaluated the expression of HIF-1α protein, a marker for hypoxia, in four groups. The expression of HIF-1α protein was increased in 5/6 (A/I) group as compared to that in Sham group, and SSR treatment for 8 weeks markedly reduced HIF-1α expression in 5/6 (A/I) rats (Figure 5(C,D)).

**Table 2.** Comparisons of COT2* and MET2* between four groups. ($\bar{x}\pm s$).

Class	Sham (*n* = 6)	5/6 (A/I) (*n* = 8)	5/6 (A/I) + LOS (*n* = 8)	5/6 (A/I) + SSR (*n* = 9)	*F*	*p* value
COT2*	54.43 ± 4.37	33.11 ± 4.95	42.60 ± 7.43	55.39 ± 5.89	25.521	< 0.0001
MET2*	35.94 ± 2.20	21.95 ± 4.28	27.15 ± 5.98	35.99 ± 3.92	19.160	< 0.0001
*t*	8.658	9.568	10.921	9.605	─	─
*p* value	0.0003	< 0.0001	< 0.0001	< 0.0001	─	─

COT2*, cortical T2*; MET2*, medullary T2*; LOS, Losartan; SSR, Shen Shuai II Recipe.

### 3.3. SSR improves renal function and reduces proteinuria in 5/6 (a/I) rats without hepatotoxicity

**Figure 6.**
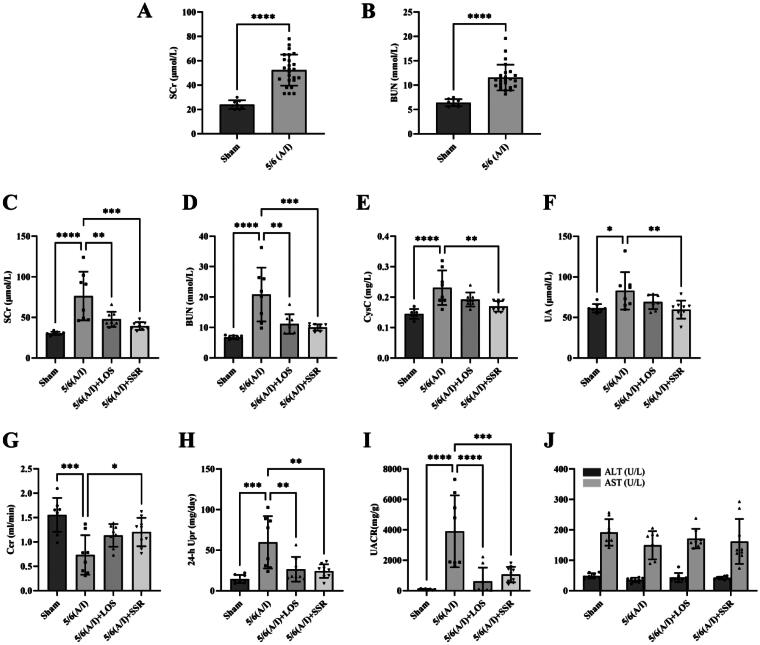
Effects of SSR on kidney and liver function and proteinuria in 5/6 (A/I) rats. The levels of SCr (A) and BUN (B) before treatment. The levels of SCr (C), BUN (D), CysC (E), UA (F), Ccr (G), 24-h Upr (H), UACR (I), ALT and AST (J) were measured after 8-week treatment. Data were analyzed by One-way ANOVA or Student’s t-test. Values are mean ± SE. *p < 0.05; **p < 0.01; ***p < 0.001; ^****^p < 0.0001. SCr, serum creatinine; BUN, blood urea nitrogen; CysC, serum cystatin C; UA, serum uric acid; Ccr, creatinine clearance rate; Upr, urinary protein; UACR, urinary albumin:creatinine ratio; ALT, alanine aminotransferase; AST, aspartate aminotransferase.

Four weeks after surgery, the levels of SCr and BUN of 5/6 (A/I) rats were significantly higher than sham-operated ones (Figure 6(A,B)), indicating that the CKD models were established successfully. After 8-week intervention, the levels of SCr, BUN, CysC, UA, 24-h Upr and UACR in the 5/6 (A/I) group were significantly higher than those in the sham operation group (all *p* < 0.05) (Figure 6(C–F,HandI)), and Ccr was significantly lower than that in the sham operation group (*p* < 0.001) (Figure 6G). However, SSR treatment significantly attenuated the increase of SCr, BUN, CysC, UA, 24-h Upr, and UACR in the 5/6 (A/I) rats, and improved Ccr (all *p* < 0.05) (Figure 6(C–1)). Furthermore, there was no significant difference in the parameters of liver function including ALT and AST among four groups (all *p* > 0.05) (Figure 6(J)). The above results show that SSR can safely improve renal function and reduce proteinuria in 5/6 (A/I) rats without hepatotoxicity.

### 3.4. SSR attenuates renal injury and fibrosis in 5/6 (a/I) rats

**Figure 7.**
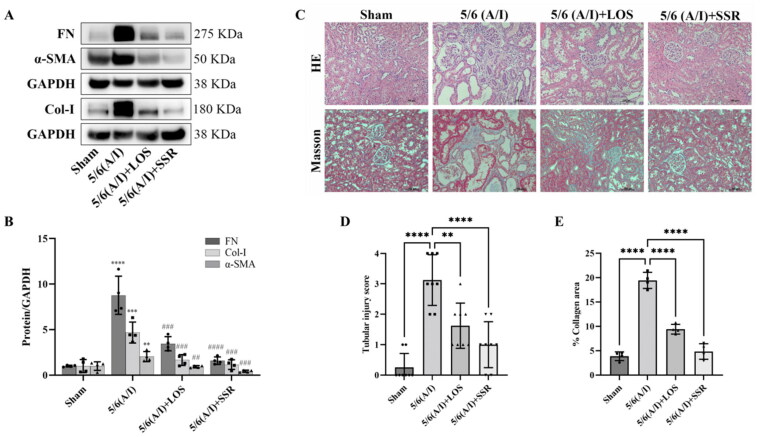
Effects of SSR on renal injury and fibrosis in 5/6 (A/I) rats. (A) The expression of FN, Col-I, and α-SMA proteins were determined by western blot. (B) Quantitative analysis of FN, Col-I, and α-SMA levels (*n* = 4). (C) Representative photomicrographs of HE and Masson’s trichrome staining. 200 × magnification. (D) The severity of tubular damage was assessed by the tubular injury score (*n* = 8). (E) Semiquantitative analysis of collagen area (*n* = 4). Data were analyzed by One-way ANOVA. Values are mean ± SE. *p < 0.05; **p < 0.01; ***p < 0.001 vs. Sham group, *^#^p* < 0.05, ^##^*p* < 0.01, ^###^*p* < 0.001 vs. 5/6 (A/I) group. LOS, Losartan; SSR, Shen Shuai II Recipe.

To investigate the effects of SSR on renal injury and fibrosis, the remnant kidneys from four groups were exerted with immunoblotting and histopathological examination (HE and Masson staining analyses). Immunoblotting results showed that the expression of FN, Col-I and α-SMA, markers of renal fibrosis, were significantly increased in 5/6 (A/I) group (Figure 7(A,B)). However, SSR treatment significantly downregulated the expression of FN, Col-I and α-SMA in 5/6 (A/I) rats (Figure 7(A,B)) (all *p* < 0.001). Furthermore, histopathological examination confirmed that the extensive renal tubular injury and fibrosis were observed in 5/6 (A/I) group compared with those in Sham group, which were attenuated by SSR treatment for eight weeks (Figure 7(C–E)).

### 3.5. Correlations of biochemical indicators with BOLD-fMRI parameters

**Figure 8.**
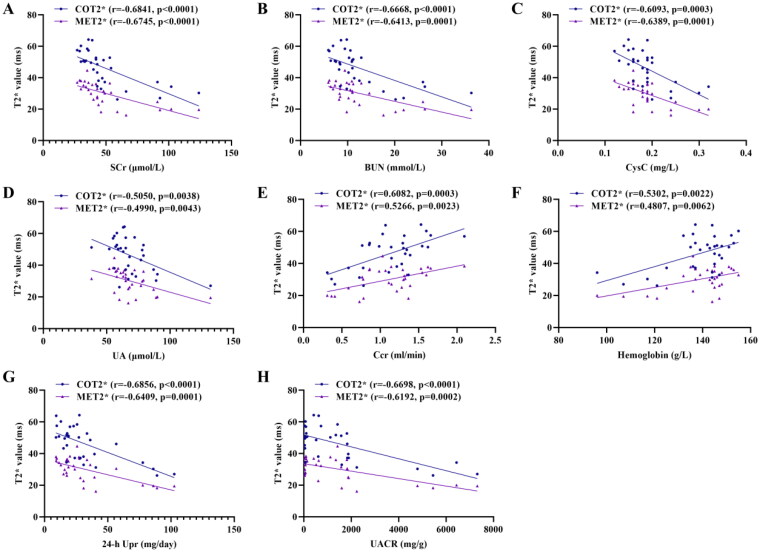
Correlation of COT2* and MET2* with SCr (A), BUN (B), CysC (C), UA (D), Ccr (E), Hemoglobin (F), 24-h Upr (G), and UACR (H). Data were analyzed by Pearson’s correlation coefficient. COT2*, cortical T2*; MET2*, medullary T2*; SCr, serum creatinine; BUN, blood urea nitrogen; Cys C, serum cystatin C; UA, serum uric acid; Ccr, creatinine clearance rate; Upr, urinary protein; UACR, urinary albumin:creatinine ratio.

Pearson correlations between the biochemical indicators and BOLD-fMRI parameters (COT2* and MET2*) were shown in Figure 8(A–H). COT2* and MET2* were significantly positive correlated with the Ccr (*r* = 0.6082, *p* = 0.0003; *r* = 0.5266, *p* = 0.0023) and hemoglobin (*r* = 0.5302, *p* = 0.0022; *r* = 0.4807, *p* = 0.0062), but negatively associated with SCr (*r* = −0.6841, *p* < 0.0001; *r* = −0.6745, *p* < 0.0001), BUN (*r* = −0.6668, *p* < 0.0001; *r* = −0.6413, *p* = 0.0001), CysC (*r* = −0.6093, *p* = 0.0003; *r* = −0.6389, *p* = 0.0001), UA (*r* = −0.5050, *p* = 0.0038; *r* = −0.4990, *p* = 0.0043) (Figure 8(A–F)). Additionally, there were negative correlation of COT2* and MET2* with 24-h Upr (*r* = −0.6856, *p* < 0.0001; *r* = −0.6409, *p* = 0.0001) and UACR (*r* = −0.6698, *p* < 0.0001; *r* = −0.6192, *p* = 0.0002) (Figure 8(G,H)).

## 4. Discussion

The global burden of CKD continues to increase, but current treatments for CKD are still limited [22]. Accumulating evidence suggests that renal hypoxia is prevalent in CKD, in despite of different initial kidney pathology, and accelerates the progression of CKD [3,23]. Improving renal oxygenation to avoid hypoxic injury may be a potential treatment for CKD. The safety and reliability of TCM has been confirmed, and considered as an effective complementary and alternative therapy for patients with CKD [24]. Several studies have shown that Chinese herbal medicines according to TCM theory could improve renal hypoxia in kidney diseases, which is related to the antioxidative, anti-inflammatory, and antifibrotic effects of herbs [25–26]. SSR, a Chinese herbal formula, has been widely used for more then 20 years and considered as a promising treatment for chronic renal failure. The safety and efficacy of SSR with the same composition and working concentrations have been demonstrated in previous studies [27–28]. Furthermore, the active components in SSR, such as salvianolic acid B from Salviae Miltiorrhizae Radix et Rhizoma, emodin from Rhei Radix et Rhizoma, berberine from Coptidis Rhizoma, have demonstrated their efficacies in the treatment of renal fibrosis [29–31]. The anti-inflammatory, antioxidant, and anti-apoptotic effects of rosmarinic acid and icariin, the main components of SSR, were also reported in previous studies [32–34].

The 5/6 (A/I) rat is a typical rodent model of CKD and has shown indirect evidence of increased oxygen consumption and intrarenal hypoxia [9,19]. In our previous study, we found that SSR could reduce renal oxygen consumption evaluated by sodium transport efficiency (QO_2_/TNa) in 5/6 (A/I) rats [9]. However, the measuring modality in that study was *in vitro* and cannot reflect the true renal tissue oxygenation in real time. BOLD-fMRI provides a noninvasive method for evaluating tissue oxygenation by deoxyhemoglobin as an endogenous marker [35]. As previously reported, BOLD-fMRI on a 3.0 T human whole-body scanner can be applied in rats when combined with a small receiver coil [36]. Therefore, a clinical 3.0 T magnetic resonance scanner with an animal-specific coil was used in this study. We selected the classical ROIs method to analyze BOLD-fMRI parameters and demonstrated the high reproducibility of ROI-based BOLD-fMRI in rat models. With the application of BOLD-fMRI, we directly quantified hypoxia in 5/6 (A/I) kidneys in the current study. The results shown that the levels of COT2* and MET2* were decreased in 5/6 (A/I) rats, and the levels of MET2* were lower than COT2* in both healthy and CKD rats, which is consistent with renal physiology and the theory of renal hypoxia in CKD [3,23]. Owing to the complex oxygen diffusion gradients and oxygen metabolic processes within kidneys, oxygenation in renal medulla is lower than that in cortex, which were confirmed by oxygen microsensors [37]. With the progression of CKD, the presence of inflammation, oxidative stress and dysregulated angiogenesis also aggravates hypoxia in both renal medulla and cortex [38–40]. Furthermore, we found that SSR could significantly improve cortical and medullary oxygenation (increase in the levels of COT2* and MET2*). In line with this, the protein expression of HIF-1α, a marker of hypoxia [41], in 5/6 (A/I) kidneys were downregulated by SSR treatment. In addition to the improvement of renal hypoxia, we also found that SSR could safely improve renal function and reduce proteinuria in 5/6 (A/I) rats, which is reflected by increased Ccr and decline in SCr, BUN, UA, CysC, 24-h Upr, and UACR without abnormalities in ALT and AST. Additionally, SSR could significantly attenuate renal fibrosis in 5/6 (A/I) rats according to immunoblotting and histopathological results. These findings demonstrate the renoprotective effects of SSR in 5/6 (A/I) rats, and suggest that the alleviation of renal injury is associated with the improvement of renal hypoxia.

In order to further explore the relationship between renal oxygenation and renal injury, we focused on correlations of biochemical indicators with T2* values. The COT2* and MET2* were positively correlated with the levels of Ccr and hemoglobin in rat models, indicating that renal oxygenation correlates with the severity of renal failure in CKD, which is consistent with our previous clinical findings [16–17]. We also found that the COT2* and MET2* were negatively associated with the levels of 24-h Upr and UACR, which provides evidence to confirm the correlation between proteinuria and renal hypoxia. There is a vicious cycle between renal hypoxia and proteinuria. Hypoxia promotes renal fibrosis and proteinuria, while excess proteinuria increases oxygen consumption of reabsorption and aggravates renal hypoxia [42].

The advantages of this study were that we first quantified renal hypoxia in 5/6 (A/I) rats using BOLD-fMRI, and demonstrated the correlation of renal oxygenation with renal function. Additionally, the study provided evidence that SSR could attenuate renal injury by improving renal hypoxia. However, the current study still has limitations. First, we have preliminarily confirmed the renoprotective effects of SSR in the treatment of CKD, but the potential mechanism and active compounds still require to be further investigated. Additionally, we noticed the potential risk of high concentration of amygdalin in SSR, but there was not clear evidence of its toxicity in this study. Perhaps the interaction among multiple components in SSR reduces the adverse effect of the single component, which requires further exploration in future studies. Second, we have assessed the diagnostic value of BOLD-fMRI in CKD models through horizontal comparison among groups, but a pretreatment evaluation of renal oxygenation state is also desirable to dynamically evaluate the changes in renal oxygenation levels before and after treatment. Third, only the 5/6 (A/I) rat model of CKD was applied in this study. More CKD models, such as unilateral ureteral obstruction model and adenine-induced CKD model, should be used in further studies to verify renal hypoxic state in CKD with diverse underlying etiologies, and confirm the diagnostic reliability of BOLD-fMRI.

## 5. Conclusions

In summary, the current study revealed that SSR could attenuate renal injury in 5/6 (A/I) rats through the improvement of renal hypoxia. Furthermore, we quantified renal oxygenation in rat models using BOLD-fMRI and confirmed the correlations between renal hypoxia and renal injury. These findings suggest that SSR could be an effective herbal formula for the treatment of hypoxic injury in CKD.

## Data Availability

The datasets generated and/or analyzed during the current study are available from the corresponding author on reasonable request.
